# Braithwaite’s Retrospect of Practical Medicine and Surgery

**Published:** 1850-03

**Authors:** 


Art. VI.—*Braithwaite's Retrospect of Practical Medicine and Surgery.* Part 20th.

*The Half-Yearly Abstract of the Medical Sciences:* Being a practical and analytical digest of the contents of the principal British and continental medical works, published during the preceding six months.—Edited by W. H. Ranking, M. D., Cantab., &c. Republished by Lindsay & Blakiston.

These periodicals still continue their half-yearly visits to the medical profession, and they are among the most useful of their class. They present a faithful record of the progress of the various departments of practical medicine, and are of great value to the practitioner; and in order that our readers may enjoy some of the advantages connected with these practical details, we shall endeavor to make an abstract of some of the most important points in Braithwaite's Retrospect.

